# The C-type lectin receptor mincle is functionally expressed by murine bone cells and can mediate inflammatory osteoblast responses to *Staphylococcus aureus*

**DOI:** 10.1016/j.bone.2025.117689

**Published:** 2025-10-25

**Authors:** Erin L. Mills, Quinton A. Krueger, Aiza Noyal, M. Brittany Johnson, Ian Marriott

**Affiliations:** a Department of Biological Sciences, University of North Carolina at Charlotte, Charlotte, NC, USA; b Computational Intelligence for Predicting Health and Environmental Risks (CIPHER), University of North Carolina at Charlotte, Charlotte, NC, USA

**Keywords:** Osteoblasts, Osteoclasts, *Staphylococcus aureus*, C-type lectin receptors, Mincle, Pattern recognition receptors, Osteomyelitis

## Abstract

It is now apparent that osteoblasts and osteoclasts have immune functions that play a critical role in shaping host responses and the abnormal bone remodeling associated with staphylococcal infections. Both cell types express various pattern recognition receptors (PRRs) that enable them to perceive pathogens and initiate the production of mediators that can exacerbate infection-induced inflammatory bone loss. Macrophage-inducible C-type lectin (Mincle) is a tyrosine activation motif–coupled PRR that can recognize glycolipids from diverse pathogens to initiate inflammatory mediator production. In the lung, an important role for Mincle has been suggested in host defense against Gram positive bacteria. In the present study, we report that RNA Tag-Seq analysis of *S. aureus* infected murine osteoclasts and osteoblasts revealed enrichment of genes associated with C-type lectin receptor-mediated responses and elevated expression of mRNA encoding Mincle and its key downstream signaling components. We have found robust levels of Mincle protein in murine osteoclasts and osteoblasts, and demonstrated the inducible expression of this molecule in primary human osteoblasts. The functional nature of Mincle expression by osteoclasts and osteoblasts was confirmed by the ability of Mincle-specific agonists to elicit inflammatory cytokine production by these cells. Importantly, we have shown that the cytokine responses of *S. aureus* challenged murine and human osteoblasts are attenuated following Mincle blockade. Together, these studies support the assertion that bone cells functionally express Mincle and that this C-type lectin can mediate, at least in part, the inflammatory immune responses of osteoblasts to *S. aureus* challenge.

## 1. Introduction

*Staphylococcus aureus* is the most common causative agent of osteomyelitis, a condition that is associated with severe inflammation and progressive bone loss. It is now apparent that bone-forming osteoblasts and bone-resorbing osteoclasts have immune functions that play a critical role in shaping host responses to this Gram-positive bacterium and the abnormal bone remodeling associated with staphylococcal infections [1]. Both osteoblasts and osteoclasts express various pattern recognition receptors (PRRs), including membrane-associated Toll-like receptors (TLRs), which enable them to perceive pathogen associated microbial patterns (PAMPs) and initiate the production of an array of soluble and cell surface molecules that can exacerbate the inflammatory bone loss commonly associated with bone infection [2–10].

Macrophage-inducible C-type lectin (Mincle) encoded by *Clec4e* is an inducible tyrosine activation motif–coupled PRR expressed on the surface of myeloid cells, such as macrophages (as reviewed in [11]). It has been found to recognize PAMPs, most often glycolipids, from pathogens as diverse as bacteria and fungi, and may even recognize mammalian damage associated molecular patterns (DAMPs), to initiate inflammatory cytokine and chemokine production [11]. In the lung, Mincle has been reported to have an important role in host defense against the Gram-positive bacteria *Streptococcus pneumoniae* via sensing of pneumococcal glycolipid glucosyl-diacylglycerol (Glc-DAG) [12]. In *S. aureus*, the bacterial cell membrane contains the glycopolymer lipoteichoic acid (LTA), which is important for bacterial evasion, adhesion, colonization, and biofilm formation [13–16], and this is anchored to Glc-DAG and another glycolipid, diglucosyl-diacylglycerol (Glc_2_-DAG) [17,18]. Interestingly, a recent study has determined that these glycolipids can exert opposing effects via Mincle in murine phagocytic myeloid cells, where Glc-DAG induces inflammatory cytokine production and Glc_2_-DAG attenuates this effect [19]. In vivo, it appears that Glc2-DAG can inhibit Glc-DAG-induced, Mincle-dependent, cytokine responses associated with *S. aureus* lung infection [19]. However, this study also indicated that sustained elevated Mincle expression can override such inhibition leading to increased susceptibility to staphylococcal pneumonia [19].

High levels of Mincle mRNA expression have been reported at dental gingival peri-implantitis sites in mice and this has been attributed to local macrophage activation [20]. Interestingly, similarly pronounced Mincle expression has been seen in human skeletal bone tissue where there are high levels of osteocyte death and osteoclast activity due to sterile fracture [21]. This suggests DAMPs may induce osteoclast activation in a Mincle-dependent manner. Such a notion is supported by the observation that murine osteoclasts express robust levels of Mincle in vitro and in vivo following exposure to necrotic osteocytes, and the demonstration that genetic deletion of this sensor improves fracture repair and attenuates associated inflammatory bone loss [21].

In the present study, we report that RNA Tag-Seq analysis of *S. aureus* infected murine osteoclasts and osteoblasts revealed enrichment of genes associated with C-type lectin receptor-mediated responses and elevated expression of mRNA encoding Mincle and its key downstream signaling components. We have found robust levels of Mincle protein in murine osteoclasts and osteoblasts, and demonstrated the inducible expression of this molecule in primary human osteoblasts. The functional nature of Mincle expression by osteoclasts and osteoblasts was confirmed by the ability of Mincle-specific agonists to elicit inflammatory cytokine production by these cells. Importantly, we have showed that the inflammatory cytokine responses of *S. aureus* challenged murine and human osteoblasts are attenuated following Mincle blockade. Together, these studies support the contention that osteoclasts and osteoblasts functionally express Mincle and that this C-type lectin can mediate, at least in part, the inflammatory immune responses of osteoblasts to *S. aureus* challenge.

## 2. Methods

### 2.1. S. aureus propagation

*S. aureus* strain UAMS-1, a clinical isolate from a patient with osteomyelitis, was grown on Luria broth (LB) agar plates overnight at 37 °C in 5 % CO_2_ and was cultured in LB on an orbital rocker at 100 rpm and 37 °C in 5 % CO_2_ overnight. Prior to use in our studies, bacteria were grown to mid-log phase in LB at 37 °C with 5 % CO_2_ and the number of colony forming units (CFU) was determined by spectrophotometry using a Genespec3 spectrophotometer (MiraiBio Inc.).

### 2.2. Isolation and culture of primary murine and human osteoblasts

Whole calvaria were isolated from two to three-day old murine neonates as we have described [2,3,22] with the following modifications. Briefly, primary osteoblasts were isolated from whole calvaria through six sequential 15-min trypsin/collagenase P digestions and the cells were maintained in DMEM supplemented with 10 % FBS and 1 % penicillin/streptomycin at 37 °C in a 5 % CO_2_ atmosphere. At 24 h, primary osteoblasts were uniformly seeded in 6-well plates at a density of 2 × 10^5^ cells per well and differentiated in αMEM supplemented with 10 % FBS, 0.1M ascorbic acid, 1M β-glycerol phosphate, and 100 Units/mL penicillin/100 μg/mL streptomycin at 37 °C in a 5 % CO_2_ atmosphere. The media was changed every other day for 10 days prior to use, whereupon the common mature differentiation status of the osteoblasts in each well was verified by assessing alkaline phosphatase levels using a commercially available staining kit (Abcam) and light field microscopy, as we have described [23].

Normal human osteoblast cultures were purchased (ScienCell, Carlsbad, CA) and propagated as described by our laboratory [24,25]. These commercially available cells have previously been characterized as being authentic osteoblasts [26].

### 2.3. Generation and culture of bone marrow derived murine osteoclasts

Primary murine osteoclasts were derived from the bone marrow of the humerus and femurs of adult male and female C57BL/6 J mice. Bone marrow cells were evenly seeded in 6-well plates and differentiated to mature osteoclasts with αMEM supplemented with 10 % FBS, 1 % penicillin/streptomycin, 100 ng/mL receptor activator of nuclear factor kappa-B ligand (RANKL) (Stem Cell Technology, Cat#78059), and 100 ng/mL macrophage colony-stimulating factor (M-CSF) (Stem Cell Technology, Cat#78059.1) for 5 days as previously described [23,27]. To confirm differentiation, tartrate-resistant acid phosphatase (TRAP) staining was performed using a commercially available kit (Sigma Aldrich) according to the manufacturer’s instructions and the presence of TRAP-positive cells was confirmed using light field microscopy [23].

### 2.4. In vitro S. aureus infection and stimulation of osteoclasts and osteoblasts

Differentiated osteoclasts and osteoblasts were infected with *S. aureus* at multiplicities of infection (MOI) that ranged between 25:1 and 150:1 bacteria to each bone cell, as indicated for each experimental series, in antibiotic-free αMEM 10 % FBS media at 37 °C with 5 % CO2 for 2 h. Following this exposure, wells were washed with sterile filtered 1× phosphate buffered saline (PBS), and then the media was replaced with αMEM, 10 % FBS, 1 % penicillin/streptomycin to kill residual extracellular bacteria. In addition, isolated cells were exposed to the Mincle agonists trehalose-6,6-dibehenate (TDB; InvivoGen SanDiego, CA) and β-glucosylceramide (BG; InvivoGen), or the TLR ligands, Pam3CSK4, peptidoglycan, and lipopolysaccharide (LPS) that were used as Mincle-independent control stimuli. At the indicated time points following bacterial infection or stimulation, cell supernatants and whole cell protein isolates were collected. The range of bacterial doses, the identity and concentration of the PRR agonists, and the experimental time points in these studies were selected based on our previously published work examining immune mediator production by osteoclasts and osteoblasts [2,22,23,27].

In some experiments, cells were treated with a mouse or human Mincle neutralizing antibody (IgG2a clone 6G5 and IgG2b clone 15H5, respectively) validated by the manufacturer for neutralization using cellular assays (InvivoGen) and as employed previously [28] or an isotype control antibody (IgG2b clone C1-12G10) (5 μg/mL) one hour prior to challenge with *S. aureus* or PRR ligands.

### 2.5. RNA sequence analysis

RNA sequence analysis was performed to assess changes in the transcriptome of murine bone marrow-derived osteoclasts and primary osteoblasts following challenge with *S. aureus*. Bone marrow derived osteoclasts and primary murine osteoblasts were infected with *S. aureus* at a bacteria-to-bone cell ratio of 75:1 and RNA was isolated using a PureLink RNA mini kit at 4 h post-infection. The Genomic Sequencing and Analysis Facility at the University of Texas at Austin prepared Tag-Seq libraries using an established method [29] and sequenced libraries using an Illumina HiSeq 3000. The 100 bp, single-end reads were trimmed and quality-filtered using the FastX-toolkit [30]. The trimmed reads were mapped to the reference genome (GRCm38.p6) using Bowtie2. Differential expression analysis was performed using R package DESeq2 [31]. Outlier analysis was performed using the ArrayQuality-Metrics R package [32]. Normalized and regularized log-transformed counts were used in all downstream analysis (R3.5.0, R Core Team, 2015) following the generation of a read-counts-per-gene file retaining only transcripts mapped to a single gene. Gene expression heatmaps were made using the PHEATMAP package clustering expression patterns hierarchically [33]. A log_2_ ratio of read counts above 1.5 was considered statistically significant (padj <0.05). Pairwise comparisons between each treatment at each timepoint using Wald tests in DESeq identified significantly differentially expressed genes (DEGs) with *p*-values calculated using the Benjamini-Hochberg procedure. False discovery rate (FDR) adjustment for multiple testing resulted in 606 DEGs. Of the 606 DEGs identified, 135 displayed greater than a 2-fold difference in expression, and p-adjusted value less than 0.05. Significant genes were characterized via gene ontology and Kyoto Encyclopedia of Genes and Genomes (KEGG), and pathway analysis conducted using ShinyGO 0.76 [34]. For this analysis, all DEGs were converted to ENSEMBL gene IDS or STRING-db protein IDs. An FDR cutoff value of 0.05 and a pathway size range of 2 to 2000 was applied. FDR was calculated based on nominal *p*-value from the hypergeometric test. The top 10 pathways were first selected by FDR and then sorted by fold enrichment. In some analyses, Chi-squared and Student’s *t*-tests were used to determine whether differentially expressed genes have special characteristics when compared with genes in the reference genome. The original datasets are available in the Gene Expression Omnibus (GEO) publicly accessible repository under the accession numbers GSE217455 and GSE287095.

In addition, we have performed a secondary analysis of a dataset available in the GEO repository under the accession number GSE166522. This study compared the transcriptome of *S. aureus* infected and uninfected murine bone tissue in an in vivo murine model of implant associated osteomyelitis over 3 and 14 days after infection [35]. The final published counts table was used as input for differential gene expression and KEGG pathway analysis as described above.

### 2.6. Immunoblot analysis of Mincle and phosphorylated Syk expression by isolated bone cells

Total cell protein isolates from osteoclasts (1 × 10^5^) and osteoblasts (1 × 10^6^) were analyzed for the presence of Mincle and pSyk by immunoblot analysis using a rabbit polyclonal antibody directed against mouse (ThermoFisher Scientific, PA5–116924) or human (Sigma-Aldrich, HPA004935) Mincle and a rabbit monoclonal antibody directed against pSyk (Cell Signaling, Cat# 44170) overnight at 4 °C. Immunoblots were then washed and incubated with an HRP-conjugated anti-rabbit secondary antibody, and bound antibody was detected with a Pierce enhanced chemiluminescence (ECL) immunoblot substrate (ThermoFisher Scientific). Immunoblots were re-probed with a rabbit polyclonal antibody against β-actin (Cell Signaling, Cat# 4967; 26 ng/mL) or tubulin (Cell Signaling, Cat# 2146; 19 ng/mL) to assess total protein loading. The immunoblots shown are representative of at least three separate experiments. Densitometric analysis was conducted using ImageLab software (BioRad) or Azure Spot Pro Software (Azure Bio-systems) and Mincle and pSyk protein levels were normalized to β-actin or tubulin expression.

### 2.7. Enzyme-linked immunosorbent assays

Specific capture ELISAs were conducted to quantify cytokine protein production in response to PRR ligand stimulation and *S. aureus* infection. Mouse IL-12/23 p40 and TNF concentrations were analyzed using commercially available ELISA DuoSet kits (R&D Systems) according to manufacturer guidelines. A rat anti-mouse (BD Biosciences, Cat# 554400; 2 μg/mL) or rat anti-human (BD Biosciences, Cat # 554543; 2 μg/mL) IL-6 capture antibody and a biotinylated rat anti-mouse (BD Biosciences, Cat# 554402; 0.05 μg/mL) or rat anti-human (BD Biosciences, Cat# 554546; 0.05 μg/mL) IL-6 detection antibody was used to quantify IL-6 levels. Streptavidin-horseradish peroxidase (HRP) (R&D Systems; Lot # P446609) was added prior to the addition of tetramethylbenzidine substrate. The colorimetric reaction was stopped with 1:30 H_2_SO_4_ and the absorbance was measured at 450 nm. Recombinant cytokines were utilized to generate standard curves and extrapolation of the absorbance to the standard curve was used to determine the concentration of each protein in the samples. The selection of the cytokine readouts was based on the species and cell type under investigation and supported by our previously published work examining immune mediator production by osteoclasts and osteoblasts [2,22,23,27,36–39].

### 2.8. Statistical analysis

Data is expressed as the mean ± standard deviation of the mean (SD). Commercially available software (GraphPad Prism, GraphPad Software, La Jolla, CA) was used to conduct statistical analyses including Student’s *t*-test, one-way analysis of variance (ANOVA) with Dunnett’s post-hoc test, or two-way ANOVA with Šídák’s multiple comparisons test, as appropriate.

## 3. Results

### 3.1. Increased expression of Mincle and associated signaling components in S. aureus infected murine bone tissue

**Fig. 1.**
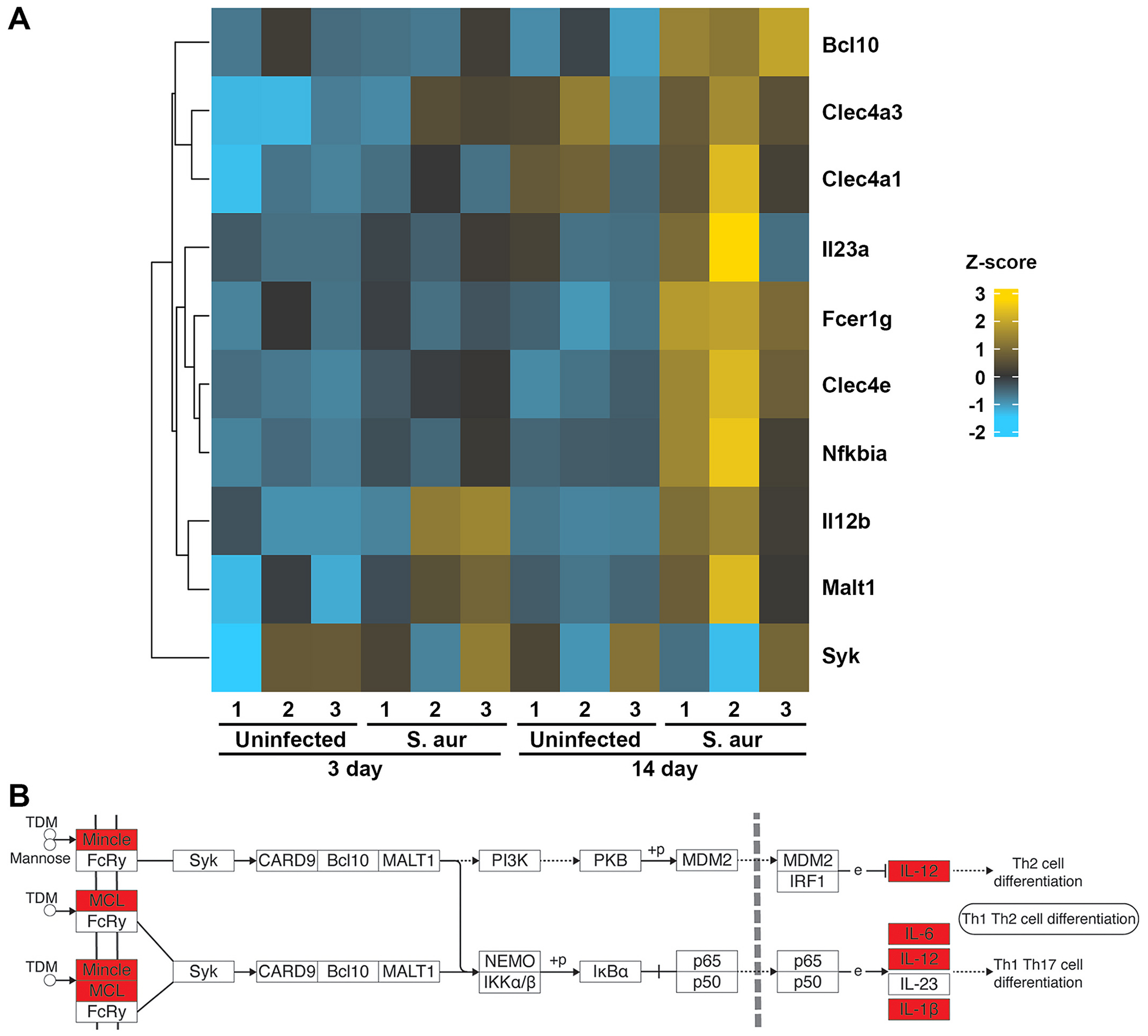
Mincle and associated signaling components show increased local mRNA expression in murine bone tissue isolated from an in vivo model of implant associated *S. aureus* infection. Lin and colleagues [35] isolated bone tissue at 3 and 14-days post-infection for transcriptional analysis, and we have performed a secondary analysis of this publicly assessable data set (accession number GSE166522). Panel A shows a heatmap displaying differential expression of genes encoding Mincle (Clec4e) and associated signaling components in uninfected and infected bone tissue (S. aur) (*n* = 3). The color key describes the range and direction of the *Z*-score. Panel B shows log_2_ fold >1.5, padj, < 0.05 upregulation of genes associated with Mincle signaling at 14-days post-infection adapted from KEGG pathway analysis of RNA Tag-Seq data conducted using ShinyGO 0.76. Enriched genes in these pathways are indicated in red. (For interpretation of the references to color in this figure legend, the reader is referred to the web version of this article.)

Examination of a published gene ontology analysis of tissue isolated from a murine model of implant associated staphylococcal osteomyelitis [35] revealed that infected bone shows enrichment of gene products associated with Mincle-mediated cellular effects following *S. aureus* challenge. Specifically, *S. aureus* infected bone tissue showed an increase in the expression of mRNA encoding Mincle, which was discernable as early as 72 h post infection and was marked at 14 days post-infection, and more modest increases in the expression of Mincle-associated signaling components, including Bcl10 and FcRγ (Fig. 1A). Furthermore, KEGG pathway analysis indicated the upregulated expression of mRNA encoding the inflammatory cytokines, IL-6 and IL-12, that have been linked to Mincle signaling in this database (Fig. 1B) [40]. While the increased level of expression of Mincle and its associated signaling components may reflect leukocyte recruitment to the infection site, it may also represent a resident bone cell response to the presence of bacteria.

### 3.2. Murine osteoclasts express Mincle and associated signaling components

**Fig. 2.**
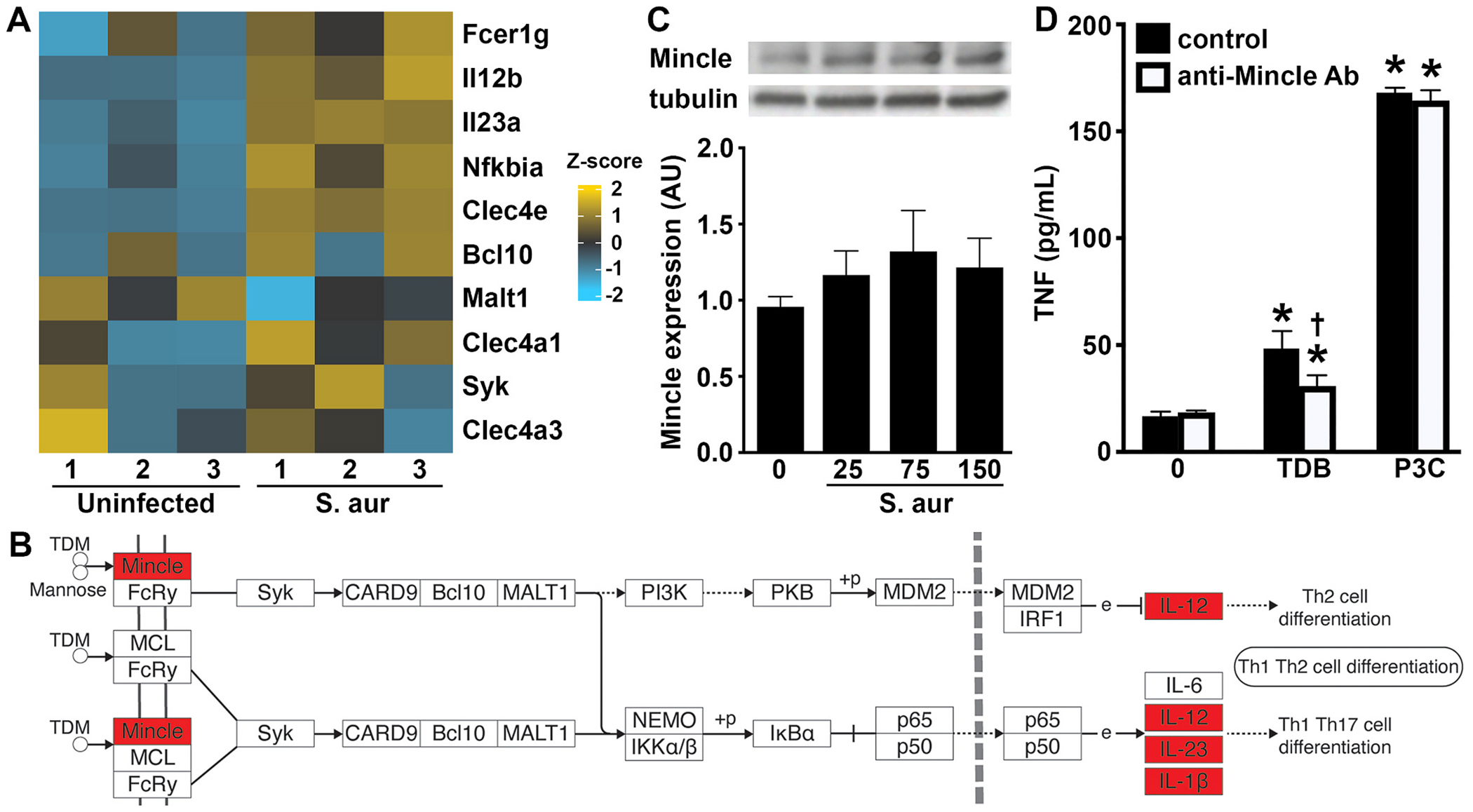
Mincle and associated signaling components are expressed in murine osteoclasts. Panels A and B: Bone marrow-derived osteoclasts were uninfected or infected with *S. aureus* at an MOI of 75:1 bacteria to each mammalian cell. At 4h, mRNA expression was assessed by RNA Tag-Seq analysis and Panel A shows a heatmap displaying differential expression of genes encoding Mincle (Clec4e) and associated signaling components in uninfected and infected osteoclasts (S. aur) (n = 3). The color key describes the range and direction of the Z-score. Panel B shows log_2_ fold >1.5, padj, < 0.05 upregulation of genes associated with Mincle signaling adapted from KEGG pathway analysis of RNA Tag-Seq data conducted using ShinyGO 0.76. Enriched genes in these pathways are indicated in red. Panel C: Osteoclasts were uninfected or infected with *S. aureus* at MOI of 25, 75, and 150 bacteria to each mammalian cell and Mincle protein levels were assessed at 8 h by immunoblot analysis. Representative immunoblots are shown and Mincle levels relative to tubulin expression are shown graphically as mean arbitrary densitometric values (AU) +/−SD (n = 3). Panel D: Osteoclasts were untreated or exposed to the Mincle agonist TDB (25 μg/mL) or the TLR2 ligand Pam3CSK4 (P3C; 10 ng/mL) in the absence or presence of a Mincle-neutralizing monoclonal antibody (anti-Mincle Ab; 5 μg/mL). At 24 h, release of TNF was assessed by specific capture ELISA. Asterisks indicate a statistically significant difference from untreated cells (0), daggers indicate statistically significant difference between control and Mincle-neutralizing antibody treated condition-matched cells (mean ± SD, n = 3; Student’s *t*-test or two-way ANOVA with Šídák’s multiple comparison test, *P* value <0.05). (For interpretation of the references to color in this figure legend, the reader is referred to the web version of this article.)

Our recent gene ontology analysis demonstrated that osteoclasts display enrichment of gene products associated with Mincle-mediated cellular effects following *S. aureus* challenge [36]. Specifically, osteoclasts showed a marked increase in the expression of mRNA encoding Mincle as early as 4 h post infection and modest increases in the expression of Mincle-associated signaling components, including Syk and Bcl10 (Fig. 2A). Furthermore, KEGG pathway analysis indicated the upregulated expression of mRNA encoding the inflammatory cytokines, IL-12 and IL-23, that have been linked to Mincle signaling in this database (Fig. 2B) [40]. Importantly, we have performed immunoblot analyses and confirmed that murine osteoclasts constitutively express Mincle protein (Fig. 2C). However, *S. aureus* challenge elicited only modest increases in Mincle protein expression levels over that seen at rest (Fig. 2C). To confirm the functional nature of Mincle expressed by osteoclasts, we have assessed the ability of TDB, a Mincle ligand [41], to elicit immune functions in these cells. As shown in Fig. 2D, this ligand elicited modest, but demonstrable, production of the key inflammatory cytokine TNF by osteoclasts. The specificity of the effect was confirmed with the demonstration that TDB-mediated TNF production by these cells was significantly reduced in the presence of a Mincle neutralizing monoclonal antibody, but this failed to inhibit TNF production mediated by a TLR ligand (Fig. 2D).

### 3.3. Mincle and associated signaling components are expressed by primary murine osteoblasts

**Fig. 3.**
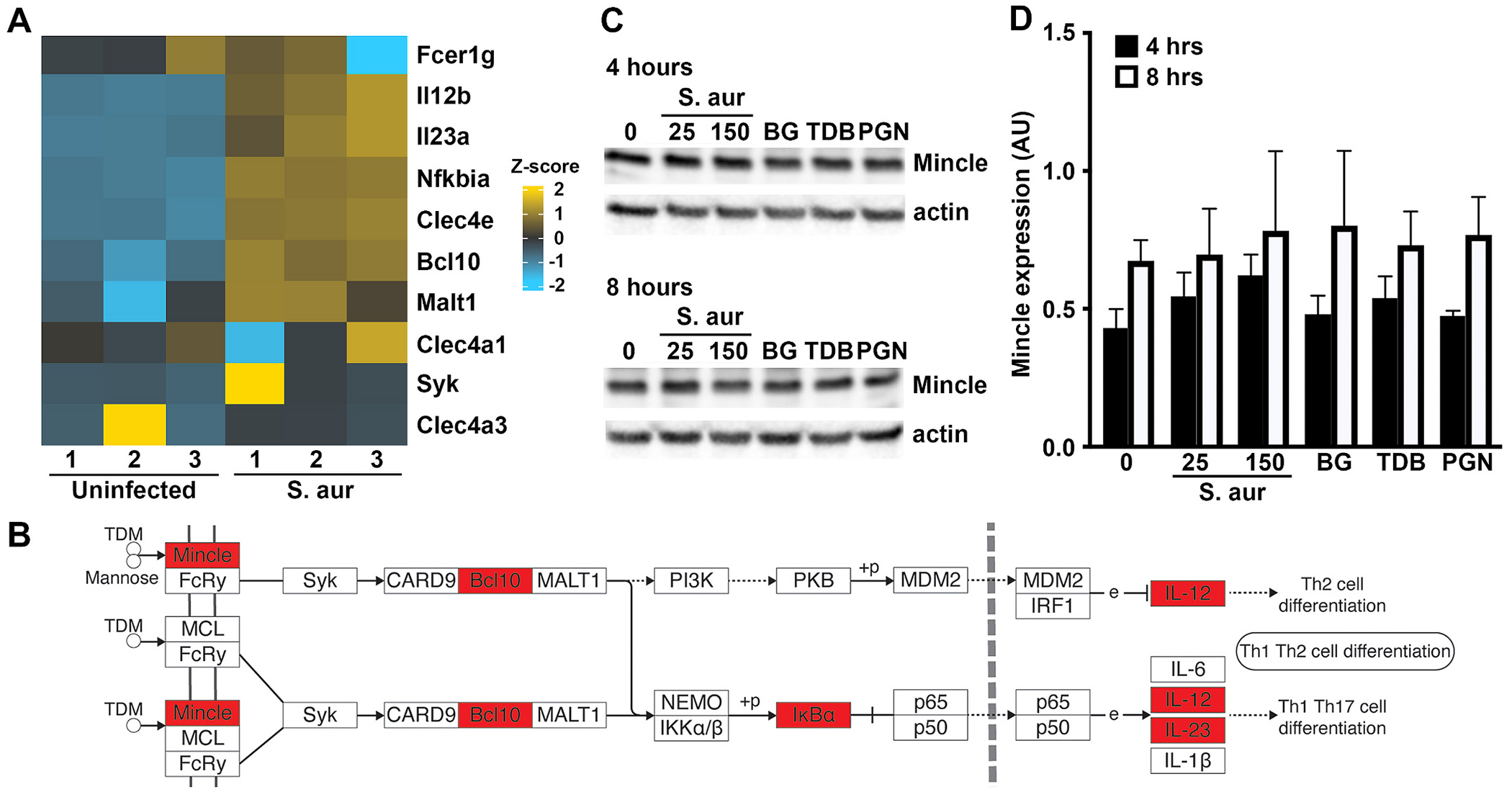
Mincle and associated signaling components are expressed in primary murine osteoblasts. Panels A and B: Osteoblasts were uninfected or infected with *S. aureus* at an MOI of 75:1 bacteria to each mammalian cell. At 4h, mRNA expression was assessed by RNA Tag-Seq analysis and Panel A shows a heatmap displaying differential expression of genes encoding Mincle (Clec4e) and associated signaling components in uninfected and infected osteoblasts (S. aur) (n = 3). The color key describes the range and direction of the Z-score. Panel B shows enrichment of genes associated with Mincle signaling, log_2_ fold >1.5, padj, < 0.05, adapted from KEGG pathway analysis of RNA Tag-Seq data conducted using ShinyGO 0.76. Enriched genes in these pathways are indicated in red. Panels C and D: Osteoblasts were uninfected, infected with *S. aureus* at MOI of 25 and 150 bacteria to each mammalian cell, or treated with the Mincle agonists BG and TDB (5 μg/mL) and the TLR2 ligand peptidoglycan (PGN; 1 μg/mL). At 4 and 8 h, Mincle protein levels were assessed by immunoblot analysis. Representative immunoblots are shown in Panel C and Mincle levels relative to actin expression are shown graphically in Panel D as mean arbitrary densitometric values (AU) (mean ± SD, n = 3; two-way ANOVA with Šídák’s multiple comparison test, P value <0.05). (For interpretation of the references to color in this figure legend, the reader is referred to the web version of this article.)

Our second recent gene ontology analysis demonstrated that primary murine osteoblasts displayed enrichment of gene products associated with Mincle-mediated cellular effects following *S. aureus* challenge [37]. Specifically, osteoblasts showed a marked increase in the expression of mRNA encoding Mincle as early as 4 h post infection (Fig. 3A). Furthermore, KEGG pathway analysis indicated the upregulated expression of mRNA encoding the Mincle-associated signaling components, Bcl10 and IκBα, and inflammatory cytokines linked to Mincle signaling, including IL-12 and IL-23 (Figs. 3A and B). Importantly, we have performed immunoblot analyses and confirmed that murine osteoblasts constitutively express robust levels of Mincle protein, which could not be elevated further by *S. aureus* challenge or exposure to the Mincle agonists TDB and BG [42], or a TLR ligand, for 4 or 8-h (Figs. 3C and D).

### 3.4. Mincle is functionally expressed by murine osteoblasts

**Fig. 4.**
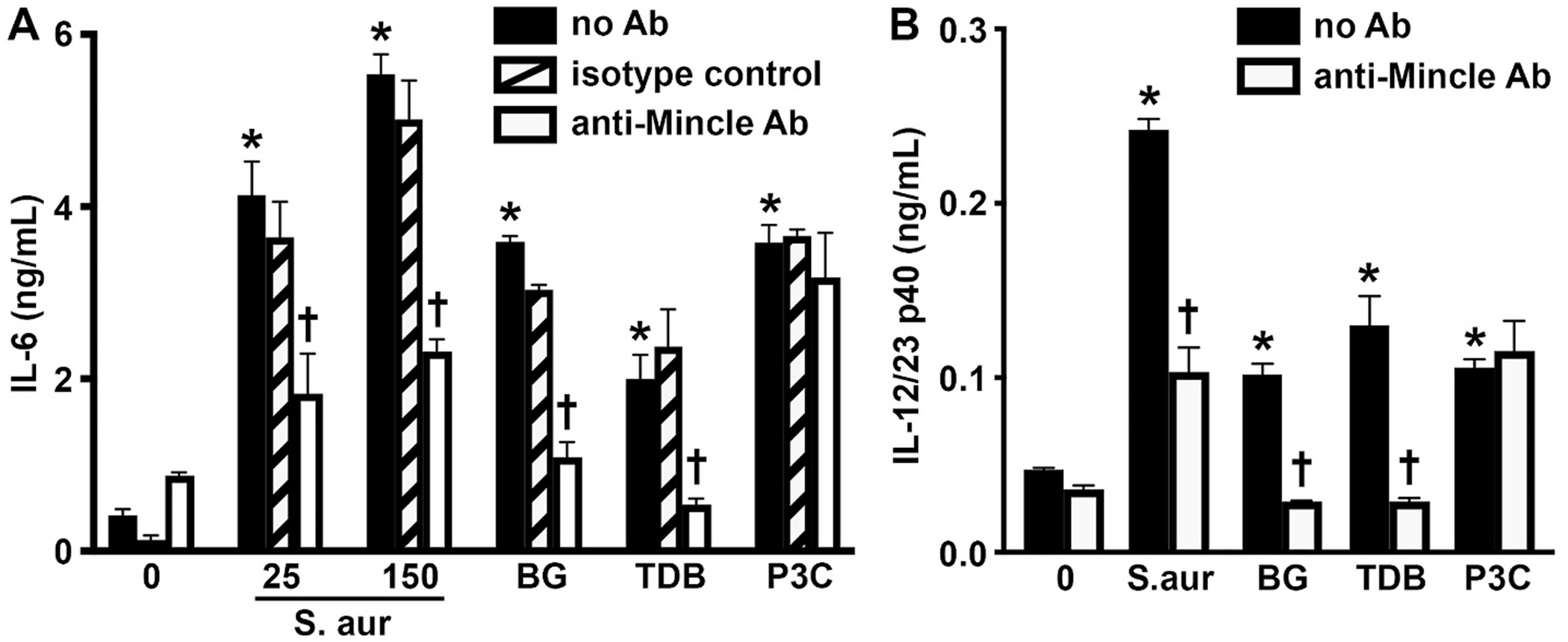
Mincle is functionally expressed by murine osteoblasts and mediates, in part, the inflammatory cytokine responses of these cells to *S. aureus* infection. Osteoblasts were untreated, exposed to the Mincle agonists BG and TDB (5 μg/mL) or the TLR2 ligand Pam3CSK4 (P3C; 10 ng/mL), or infected with *S. aureus* at MOI of 25 and 150 bacteria (Panel A) or 150 bacteria (Panel B) to each mammalian cell, in the absence or presence of a Mincle-neutralizing monoclonal antibody (anti-Mincle Ab; 5 μg/mL) or an isotype control antibody. At 8 h, release of IL-6 (Panel A) and IL-12/23 p40 (Panel B) was assessed by specific capture ELISA. Asterisks indicate a statistically significant difference from uninfected cells (0), daggers indicate statistically significant difference between control and Mincle-neutralizing antibody treated condition-matched cells (mean ± SD, n = 3; two-way ANOVA with Šídák’s multiple comparison test, P value <0.05).

To begin to establish the functional nature of Mincle expression by murine osteoblasts we have assessed the ability of two Mincle ligands, BG and TDB, to elicit the immune functions of these cells. As shown in Fig. 4A, both of these ligands elicited the production of the key inflammatory cytokine IL-6 by osteoblasts. Furthermore, both of these Mincle ligands induced the release of the p40 subunit of IL-12 and IL-23 (Fig. 4B), consistent with the activation of the Mincle signaling pathway shown in Fig. 3B. The specificity of the actions of these agonists was confirmed with the demonstration that the presence of a Mincle-neutralizing monoclonal antibody, but not an isotype control antibody, abolished BG and TDB-mediated production of IL-6 and IL-12/23 p40 but failed to inhibit the production of either of these cytokines elicited by a TLR ligand (Fig. 4).

### 3.5. The inflammatory responses of murine osteoblasts to S. aureus are mediated, in part, by Mincle

The ability of *S. aureus* infection to elevate murine osteoblast expression of mRNA encoding Mincle and associated signaling components (Fig. 3A and B), and the observation that exposure to *S. aureus* elicits the activation of Syk in these cells (Supplemental Fig. S1A), is suggestive of a role for this PRR in their responses to bacterial challenge. To more definitively assess the relative importance of Mincle in osteoblast responses to bacterial infection, we have determined the effect of a Mincle-neutralizing antibody on inflammatory cytokine production by these cells following *S. aureus* challenge. As shown in Fig. 4, *S. aureus* infection elicits the robust production of IL-6 and IL-12/23 p40 by primary murine osteoblasts. Importantly, Mincle neutralization significantly attenuated the release of both of these cytokines following *S. aureus* infection. The specificity of this effect was confirmed with the demonstration that an isotype control antibody had no effect on *S. aureus* induced osteoblast responses (Fig. 4A), and with the observation that Mincle neutralization had no effect on TLR2 ligand-induced production of either IL-6 or IL-12/23 p40 (Fig. 4). Interestingly, neither Mincle agonist nor a TLR ligand had an effect on intracellular bacterial burden following *S. aureus* infection (Supplemental Fig. S1B).

### 3.6. Mincle is constitutively and inducibly expressed by primary human osteoblasts

**Fig. 5.**
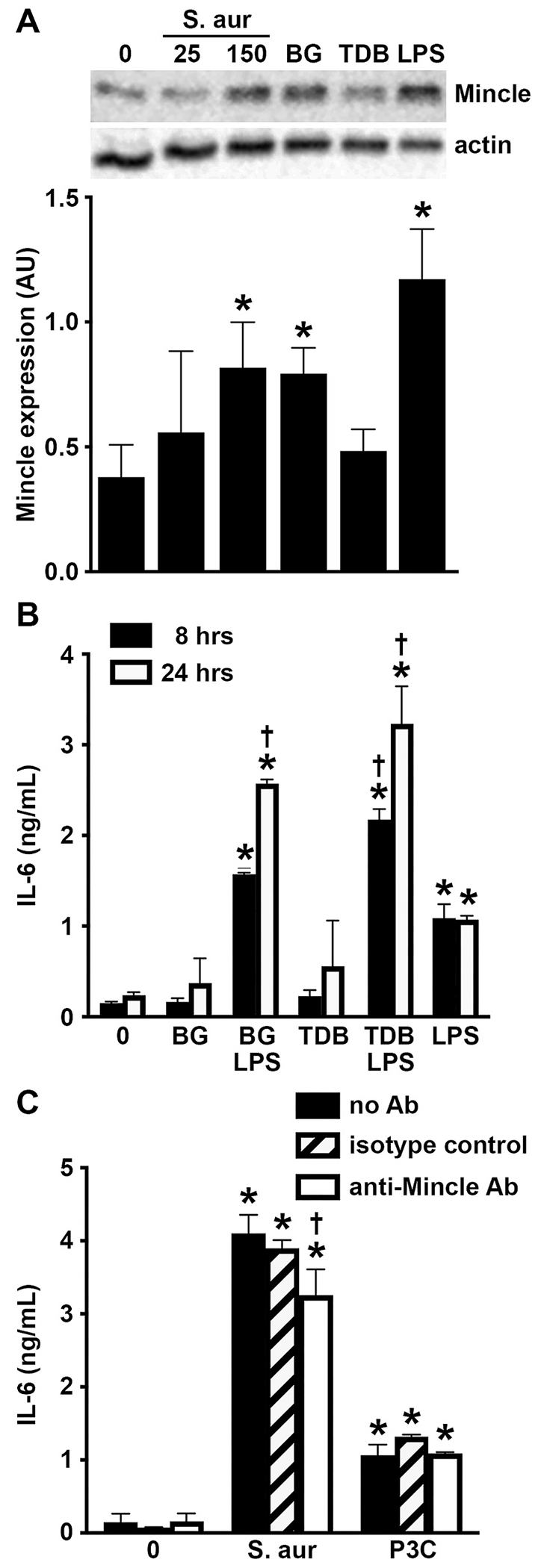
Mincle is constitutively and inducibly expressed by primary human osteoblasts. Panel A: Human osteoblasts were uninfected or infected with *S. aureus* at MOI of 25 and 150 bacteria to each mammalian cell, or treated the Mincle agonists BG and TDB (50 μg/mL) or a TLR4 ligand (LPS; 10 ng/mL). At 8 h, Mincle protein levels were assessed by immunoblot analysis. A representative immunoblot is shown in Panel A and Mincle levels relative to actin expression are shown graphically as mean arbitrary densitometric values (AU) +/− SD (*n* = 4). Asterisks indicate a statistically significant difference from unchallenged cells (0) (*p* < 0.05). Panel B: Human osteoblasts were untreated or exposed to LPS (0.5 ng/mL) 2 h prior to challenge with BG or TDB (10 μg/mL). At 8 and 24-h following challenge, release of IL-6 was assessed by specific capture ELISA. Asterisks indicate a statistically significant difference from time-matched unchallenged cells (0), daggers indicate statistically significant difference between control and LPS-treated condition-matched cells (mean ± SD, n = 3; Student’s *t*-test or two-way ANOVA with Šídák’s multiple comparison test, P value <0.05). Panel C: Human osteoblasts were untreated (0), exposed to the TLR2 ligand Pam3CSK4 (P3C; 10 ng/mL), or infected with *S. aureus* at an MOI of 150 bacteria to each mammalian cell, in the absence or presence of a Mincle-neutralizing monoclonal antibody (anti-Mincle Ab; 5 μg/mL). At 24 h, release of IL-6 was assessed by specific capture ELISA. Asterisks indicate a statistically significant difference from untreated cells, daggers indicate statistically significant difference between control and Mincle-neutralizing antibody treated condition-matched cells (mean ± SD, n = 3; two-way ANOVA with Sídák’s multiple comparison test, P value <0.05).

To begin to establish the clinical relevance of Mincle expression beyond murine bone cells, we have assessed the ability of primary human osteoblasts to express this PRR. As shown in Fig. 5A, resting human osteoblasts possess only low levels of Mincle. However, stimulation of these cells with the Mincle agonist BG or a TLR ligand induced significant elevations in Mincle levels, and *S. aureus* challenge similarly elicited dose dependent increases in the expression of this PRR. To begin to assess the functional nature of such expression by human osteoblasts, we assessed the ability of Mincle ligands to elicit immune functions in these cells. Interestingly, neither BG nor TDB alone induced statistically significant IL-6 production by human osteoblasts (Fig. 5B). However, human osteoblasts were responsive to the presence of Mincle ligands following 2-h pretreatment with LPS (0.5 ng/mL), with both BG and TDB eliciting significantly greater IL-6 responses than LPS alone at 24 h (Fig. 5B), consistent with the ability of this TLR ligand to elevate Mincle protein expression in these cells.

To begin to establish the importance of Mincle in human osteoblast responses to bacterial infection, we again investigated the effect of a Mincle neutralizing antibody on inflammatory cytokine production by these cells following *S. aureus* challenge. As shown in Fig. 5C, *S. aureus* infection elicited the robust production of IL-6 by primary human osteoblasts but such responses were reduced followed Mincle neutralization. The specificity of this effect was confirmed with the observations that an isotype control antibody had no effect on *S. aureus*-induced IL-6 production and Mincle-neutralizing monoclonal antibody treatment had no effect on IL-6 release elicited by a TLR ligand (Fig. 5C).

## 4. Discussion

The C-type lectin receptor Mincle is expressed by myeloid immune cells and functions as a transmembrane PRR that can recognize both PAMPs for host defense and DAMPs released from dead/dying cells (as reviewed in [43]). Following ligation, signaling via this cell surface sensor precipitates the production of inflammatory mediators that could serve either to promote protective immunity or exacerbate detrimental inflammation [11,43]. In the present study, we have confirmed that osteoclasts express Mincle mRNA and protein constitutively in keeping with the monocytic origin of this cell type. Interestingly, we have found that levels of mRNA encoding Mincle were significantly elevated following challenge with *S. aureus*, a finding that is consistent with a prior demonstration showing that osteoclasts show robust Mincle expression following DAMP exposure [21]. Importantly, we have shown that such expression is functional by demonstrating the ability of a known Mincle ligand to elicit immune mediator production by these cells, albeit at modest levels compared to TLR-mediated activation. Again, these results are consistent with the previous reports of DAMP-associated Mincle signaling and oxidative activity in murine osteoclasts [21].

In contrast to osteoclasts, osteoblasts are of mesenchymal lineage, but we and others have demonstrated their ability to respond to *S. aureus* challenge by producing an array of inflammatory and pro-osteoclastogenic mediators [3–5]. Furthermore, we have described the expression of various cytosolic and cell surface PRRs by murine and human osteoblasts that can underlie such responses [2–5,38,39]. Here, RNA Tag-Seq analysis of *S. aureus* infected murine osteoblasts revealed enrichment of genes associated with C-type lectin receptor-mediated responses and elevated expression of mRNA encoding Mincle and its key downstream signaling components, and immunoblot analysis showed robust levels of Mincle in both resting and bacterially challenged cells. The functional status of such expression was established by the ability of known Mincle ligands to elicit osteoblast production of inflammatory cytokines, such as IL-12/23, that are linked to Mincle signaling pathways as shown in Fig. 2C, and the specificity of these effects was confirmed with their abolition following Mincle blockade. In addition, we have performed studies to establish the relevancy of these findings to human bone cells. Interestingly, we found that human osteoblasts express only low levels of Mincle constitutively, and we showed that these resting cells failed to consistently respond to Mincle agonists. However, we noted that Mincle expression was significantly elevated in human osteoblasts following *S. aureus* challenge or activation via ligation of other PRRs. Such upregulated Mincle expression could, therefore, underlie our demonstration that ligands for this molecule can significantly augment TLR agonist-mediated human osteoblast inflammatory cytokine responses.

An important host defense role for Mincle has been reported in the lung against *S. aureus* and another clinically important Gram-positive bacterium, *Streptococcus pneumoniae* [12,19]. In these cases, Mincle recognizes the common glycolipid, Glc-DAG, to elicit inflammatory mediator production by murine phagocytic myeloid cells [12,19]. It should be noted that *S. aureus* possesses a second glycolipid attached to cell membrane LTA, Glc_2_-DAG [17,18], and this has been reported to exert an opposing inhibitory effect on Mincle-dependent inflammation in *S. aureus* lung infection [19]. However, this study also indicated that sustained elevated Mincle expression can override such an inhibition leading to an increased susceptibility to staphylococcal pneumonia [19]. Here, we have analyzed a publicly accessible database and found that the expression of mRNA encoding Mincle and associated signaling components are upregulated in *S. aureus* infected bone tissue from a murine implant associated osteomyelitis model [35]. Furthermore, we have demonstrated the elevated expression of Mincle mRNA and protein and associated signaling components in osteoblasts following exposure to *S. aureus*, and the ability of this bacterium to elicit Syk phosphorylation in these cells, implicating this PRR in bone cell responses to infection. More compelling evidence for such a role comes from the demonstration that the inflammatory cytokine responses of murine and human osteoblasts to *S. aureus* challenge, but not those due to TLR-mediated activation, are significantly attenuated following Mincle blockade.

Presumably, *S. aureus* is recognized by Mincle following its initial interaction with the surface of bone cells, but it is also possible that this PRR could continue to elicit cellular responses if this membrane associated receptor is internalized as these organisms employ the “zipper” mechanism of cell invasion [44–46]. Interestingly, we have found that Mincle-mediated osteoblast activation had no effect on the number of viable *S. aureus* harbored by these cells following infection, and a ligand for another cell surface PRR, TLR2, similarly failed to impact intracellular bacterial burden. Such findings are in contrast with our recent studies demonstrating that osteoblast activation via the cytosolic PRRs, cGAS and RIG-I, significantly reduces the number of viable bacteria harbored within these cells [27,39]. This disparity is likely due to the ability of these cytosolic sensors to trigger type I IFN responses, which we have shown can impact intracellular *S. aureus* survival/replication in osteoblasts [27,39], while responses via such surface PRRs do not [39].

Together, the present findings support the assertion that the inflammatory responses of bone cells to *S. aureus* infection can occur, at least in part, via Mincle in a similar manner to that seen for other cell types at anatomical sites including the lungs. It should be noted that while the present in vitro approaches permit the controlled investigation of Mincle in bone cell responses to bacterial challenge, such model systems do not consider interactions with infiltrating cells or bone cell-cell interactions that are likely to occur in vivo. Furthermore, it remains to be determined whether Mincle-mediated bone cell responses serve to promote appropriate host defenses to mitigate infection or, alternatively, exacerbate the inflammatory bone loss associated with staphylococcal osteomyelitis.

## Supplementary Material

Supplemental data

Raw data

Supplementary data to this article can be found online at https://doi.org/10.1016/j.bone.2025.117689.

## Data Availability

Access to raw data will be available upon reasonable request after publication. The original RNA Tag-Seq datasets are available in the Gene Expression Omnibus (GEO) publicly accessible repository under the accession numbers GSE217455 and GSE287095.
