# Supplementary figures and images for: The C-type lectin receptor mincle is functionally expressed by murine bone cells and can mediate inflammatory osteoblast responses to Staphylococcus aureus
Source: Bone. Author manuscript; Available in PMC 2026 May 19. (PMC13186104; doi:10.1016/j.bone.2025.117689)

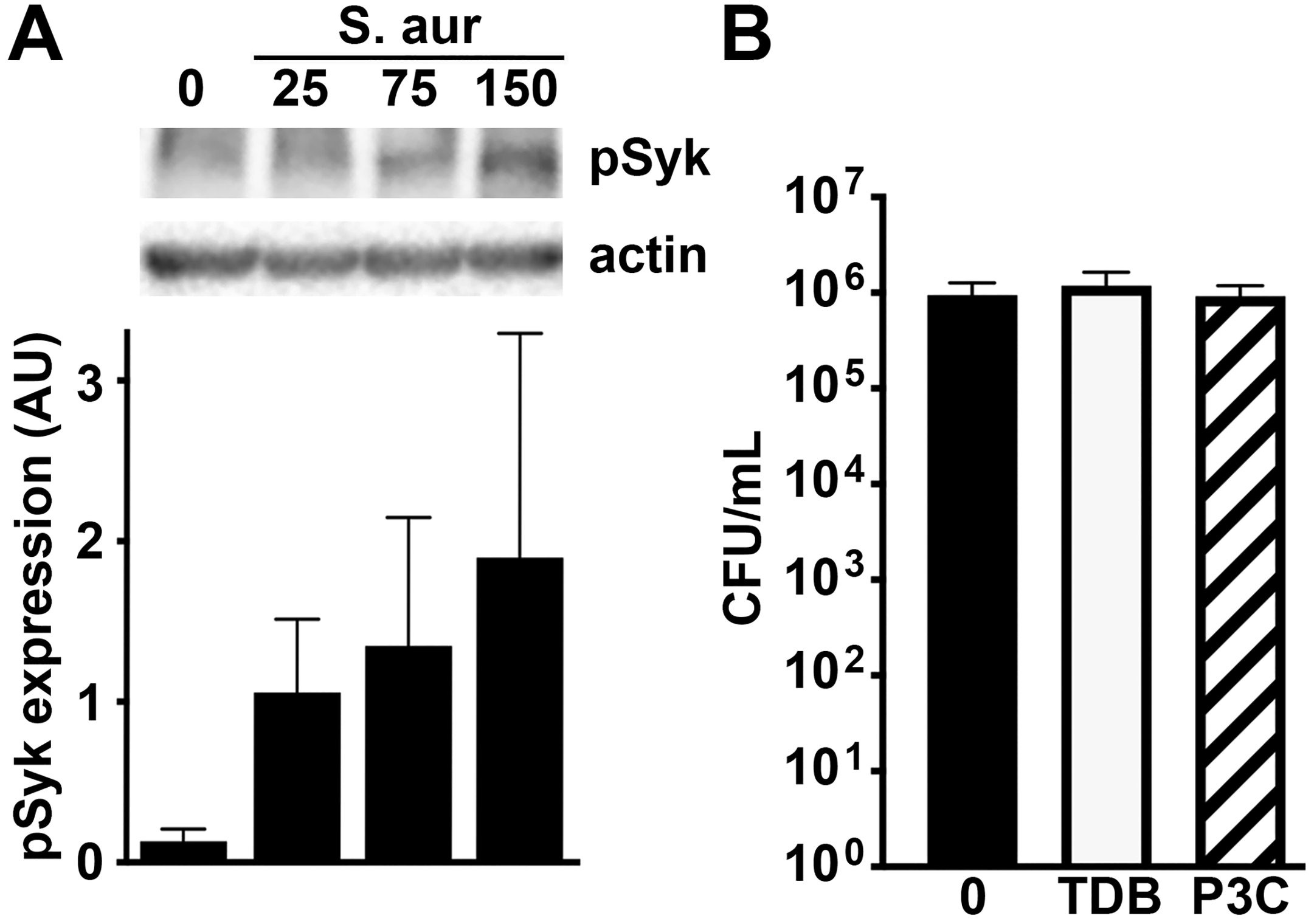

Supplement: Supplemental data [file NIHMS2155866-supplement-Supplemental_data.jpg]
