# Supplementary material for: The C-type lectin receptor mincle is functionally expressed by murine bone cells and can mediate inflammatory osteoblast responses to Staphylococcus aureus
Source: Bone. Author manuscript; Available in PMC 2026 May 19. (PMC13186104; doi:10.1016/j.bone.2025.117689)
Supplement: Raw data [file NIHMS2155866-supplement-Raw_data.pdf]

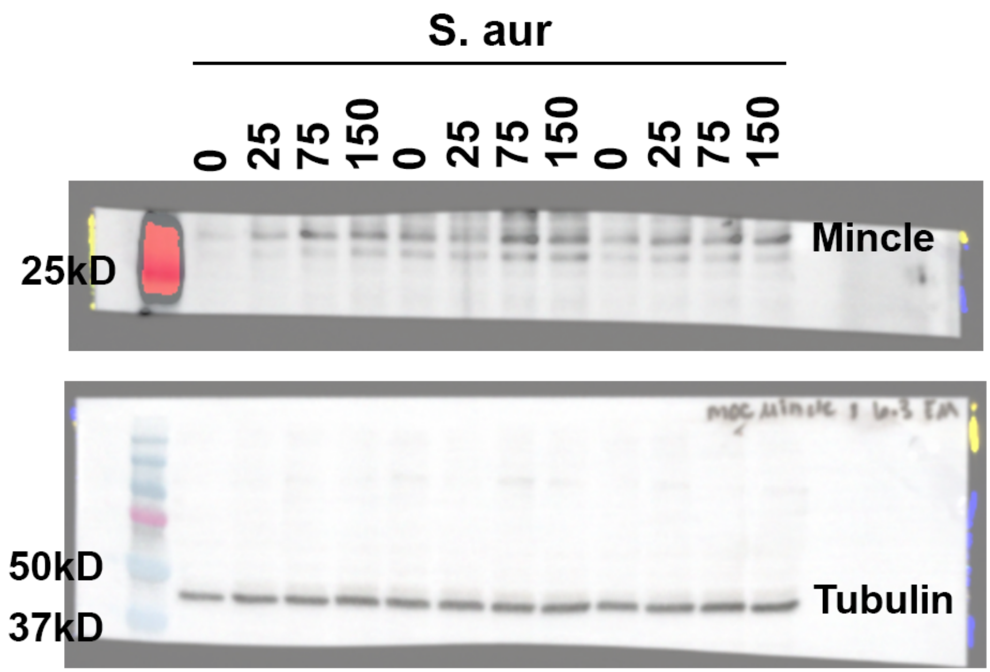

FIGURE 2C full blots

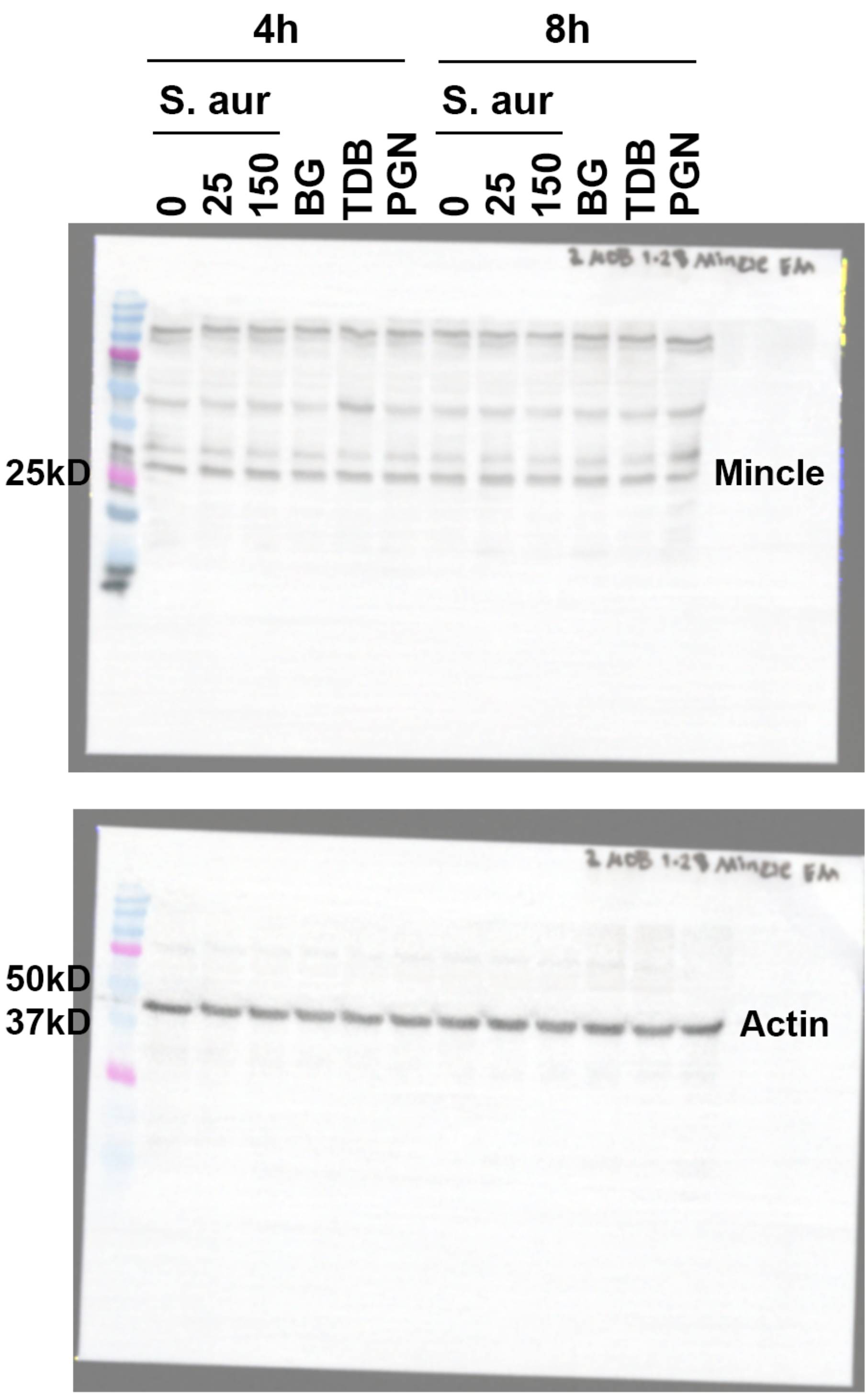

FIGURE 3C full blots

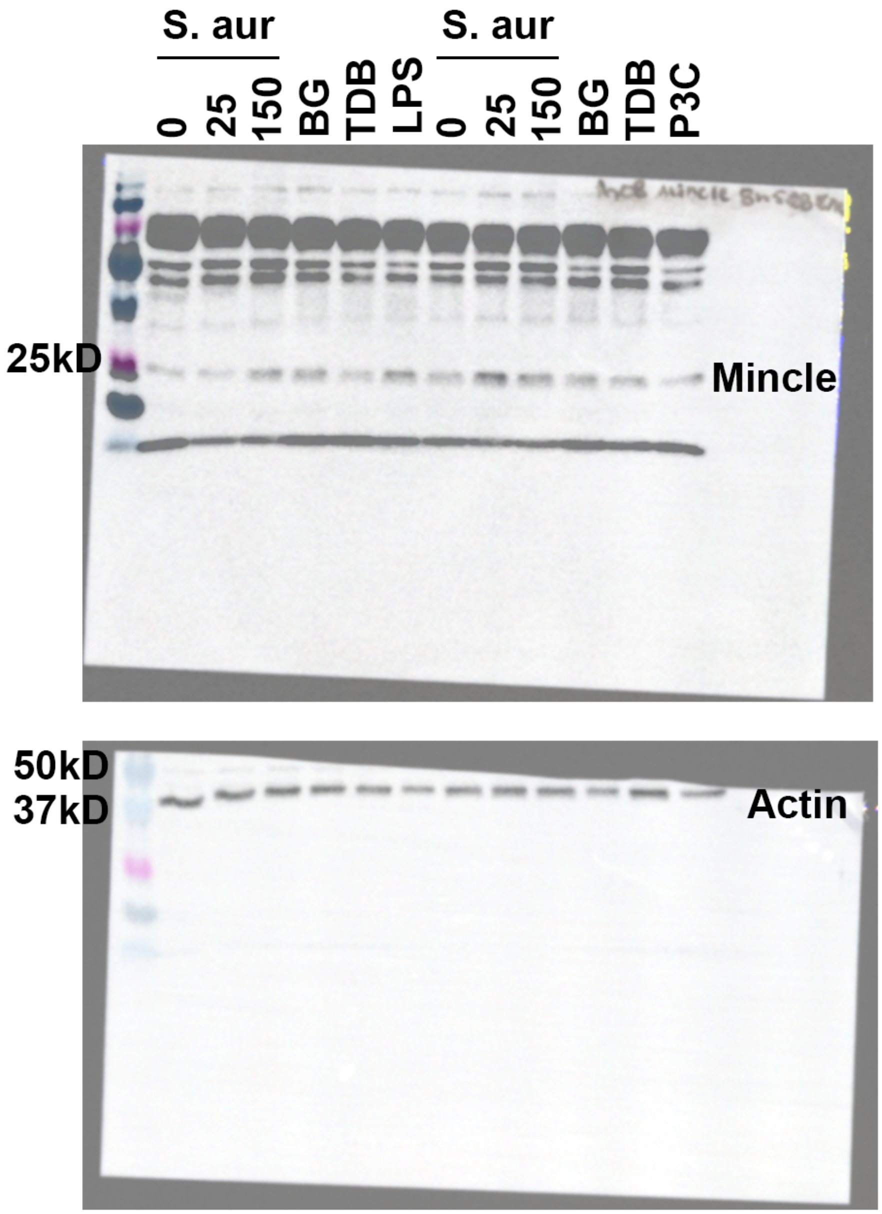

FIGURE 5A full blots

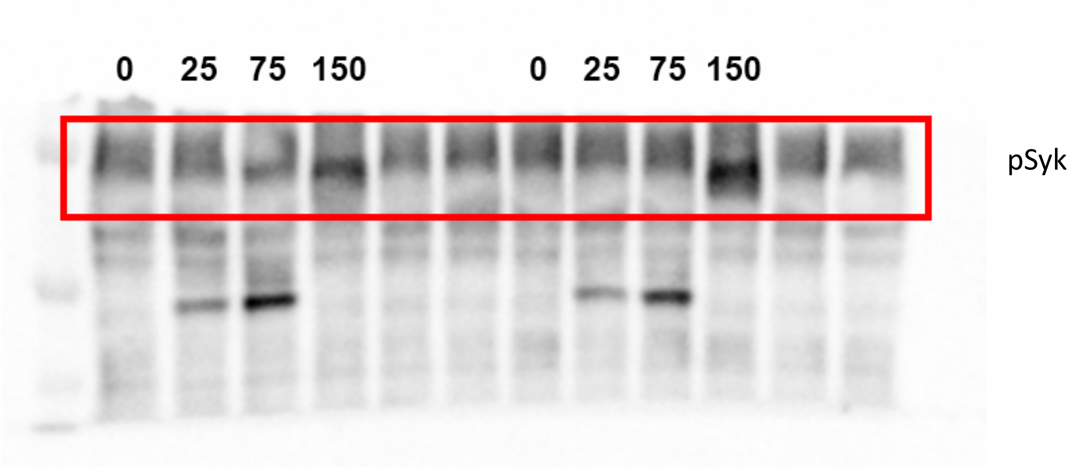

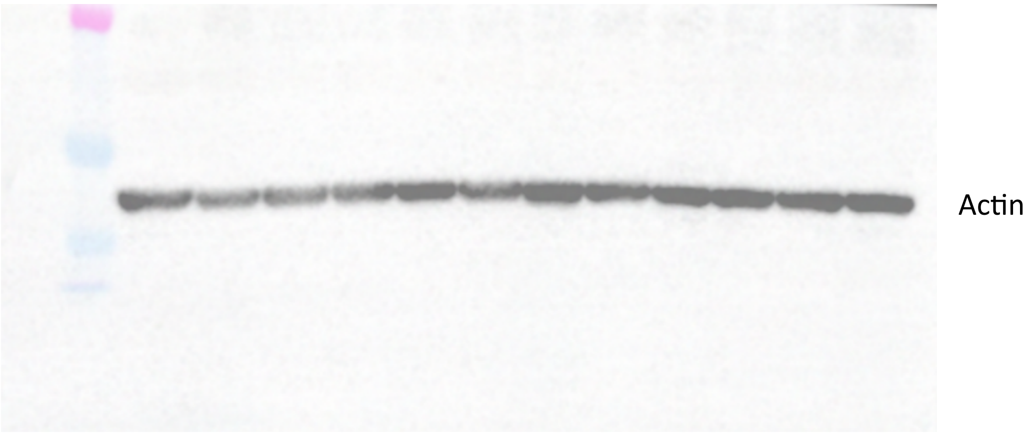

SUPPLEMENTAL FIGURE S1A full blots (set to left used in figure)
